# Temporal and sequence-related variability in diffusion-weighted imaging of presumed cerebrovascular accidents in the dog brain

**DOI:** 10.3389/fvets.2022.1008447

**Published:** 2022-11-07

**Authors:** Elizabeth Boudreau, Sharon C. Kerwin, Emily B. DuPont, Jonathan M. Levine, John F. Griffin

**Affiliations:** ^1^Department of Small Animal Clinical Sciences, School of Veterinary Medicine and Biomedical Sciences, Texas A&M University, College Station, TX, United States; ^2^Department of Large Animal Clinical Sciences, School of Veterinary Medicine and Biomedical Sciences, Texas A&M University, College Station, TX, United States

**Keywords:** diffusion, ADC, ischemia, stroke, canine, MRI

## Abstract

Diffusion-weighted MRI (DWI) is often used to guide clinical interpretation of intraparenchymal brain lesions when there is suspicion for a cerebrovascular accident (CVA). Despite widespread evidence that imaging and patient parameters can influence diffusion-weighted measurements, such as apparent diffusion coefficient (ADC), there is little published data on such measurements for naturally occurring CVA in clinical cases in dogs. We describe a series of 22 presumed and confirmed spontaneous canine CVA with known time of clinical onset imaged on a single 3T magnet between 2011 and 2021. Median ADC values of < 1.0x10^−3^ mm^2^/s were seen in normal control tissues as well as within CVAs. Absolute and relative ADC values in CVAs were well-correlated (R^2^ = 0.82). Absolute ADC values < 1.0x10^−3^ mm^2^/s prevailed within ischemic CVAs, though there were exceptions, including some lesions of < 5 days age. Some lesions showed reduced absolute but not relative ADC values when compared to matched normal contralateral tissue. CVAs with large hemorrhagic components did not show restricted diffusion. Variation in the DWI sequence used impacted the ADC values obtained. Failure to identify a region of ADC < 1.0x10^−3^ mm^2^/s should not exclude CVA from the differential list when clinical suspicion is high.

## Introduction

The assessment of restricted diffusion in brain tissue *via* MRI is used routinely for evaluation of suspected cerebrovascular accidents (CVA) in human neuroimaging (1, 2). While restricted diffusion is not pathognomonic for CVAs, it can be used to evaluate the extent of injury (3, 4), including identification of clinically silent regions of injury (5, 6), and to track the fate of injured tissue (7, 8). It is well-established that the diffusion-weighted imaging (DWI) characteristics in human CVA change over time (9–13).

Although certain diffusion-weighted characteristics [i.e., median apparent diffusion coefficient (ADC)] have been reported for a small number of CVAs in dogs (14–16), most clinical canine DWI studies have focused on neoplastic (17, 18) or inflammatory lesions (19, 20), or on diffusion tensor tractography (21–24).

The relationship between lesion age and MRI appearance of CVA in the dog brain has been described in experimental studies with induced lesions, including the temporal evolution of their appearance on DWI (24–30), but these have rarely focused on the acute to early subacute (24 h−7 d) phases of injury during which most veterinary patients with suspected CVA would be expected to have MRI performed. Also, it is not known if induced lesions are representative of the changes seen after naturally occurring CVA.

Temporal evolution of the DWI appearance of CVA is different between species (4, 9, 31). Therefore, it is not sufficient to assume that the diffusion-weighted appearance of naturally occurring canine CVA will be the same as that in humans or rodents, for whom more robust data sets are already available.

Expected changes in diffusion-weighted signal may be affected by the presence of blood, especially with echoplanar (EPI) DWI techniques that are more sensitive to susceptibility artifact. Since hemorrhagic transformation of infarction is a well-known complication in human CVA (32, 33) and is thought to occur in dogs (34, 35), understanding of the DWI appearance of both hemorrhagic and ischemic CVA is necessary for correct interpretation in a veterinary clinical setting.

We report on the DWI characteristics of presumptive spontaneous canine CVA as they relate to the age of the insult, the type of DWI (EPI vs. non-EPI) and the presence or absence of a hemorrhagic component of the lesion for a series of cases imaged at a single institution on a single 3T magnet between 2011 and 2021.

## Materials and methods

### Case identification

A single institution's medical record database was reviewed for cases meeting the predetermined inclusion criteria. To be included, dogs must have had a complete standard brain imaging series, including T2-weighted fluid attenuated inversion recovery (T2-FLAIR) imaging, T2^*^-weighted imaging, and DWI in the transverse plane, performed on a single 3T magnet (Siemens Verio). Sequence parameters for the standard brain imaging series are included in Supplementary Table S1. Eligible cases had MRI performed between July 2011 and December 2021. Identified cases were imaged using either an EPI (Siemens proprietary RESOLVE) or a non-EPI (Siemens proprietary BLADE) DWI sequence. Supplementary Table S1 contains details of these sequences for our institution. The b-values for the two sequences were selected based on visual optimization on past clinical case data by radiology faculty and technical staff and were not adjusted during the collection of the cases in this report. Also see the Discussion for further comparison of the two sequences.

Included cases were further required to meet the following requirements: 1) a solitary intra-axial lesion identified at the time of original MRI interpretation with CVA listed as a possible differential diagnosis (regardless of its position on the prioritized differential list); 2) no confirmed alternative diagnosis nor definitive treatment for another presumed diagnosis (i.e., immunosuppression, radiation therapy, chemotherapy, or surgery). All identified cases also had 3D pre- and post-Gadolinium T1-weighted sequences performed, though these were not required for inclusion nor analyzed in this report.

To be retained, cases were required to have at least one of the following: ≥5 months clinical follow-up with no progression of signs; histopathological confirmation of the nature of the lesion; or repeat MRI showing a static to resolving lesion with no progression of clinical signs in the interim. Additionally, cases were excluded if the lesion was < 5 mm in its largest dimension due to resolution limitations on the diffusion-weighted images.

The onset of signs was required to be acute (< 48 h from normal to affected). The ages of the lesions were measured as the time between onset of signs documented in the electronic medical record and time of MRI.

### Definition of DWI and ADC region of interest (ROI)

A freehand ROI tool was used to outline the volume of the region hyperintense to contralateral parenchyma on T2-FLAIR transverse images for all lesions and the volume of the region hypointense to contralateral parenchyma on T2^*^-weighted transverse images for hemorrhagic lesions. These volumetric ROIs were then copied to the corresponding DWI and ADC images. For comparison, an ellipsoidal volume in a corresponding contralateral parenchymal region was similarly selected (OsiriX version 12.5.2). The individual pixel values within the regions of interest were extracted for descriptive statistical analysis (Matlab Mac R2022a). Both raw ADC values and the ratio of ADC values to the median value of the contralateral control region are reported. Regions of interest were drawn on anonymized images by a single boarded neurologist/radiology resident (EBM) blinded to all clinical information.

Due to the small number of cases and numerous sources of variability in this retrospective case series, formal statistical analysis of these data was deemed inappropriate.

## Results

### Case selection

**Figure 1:**
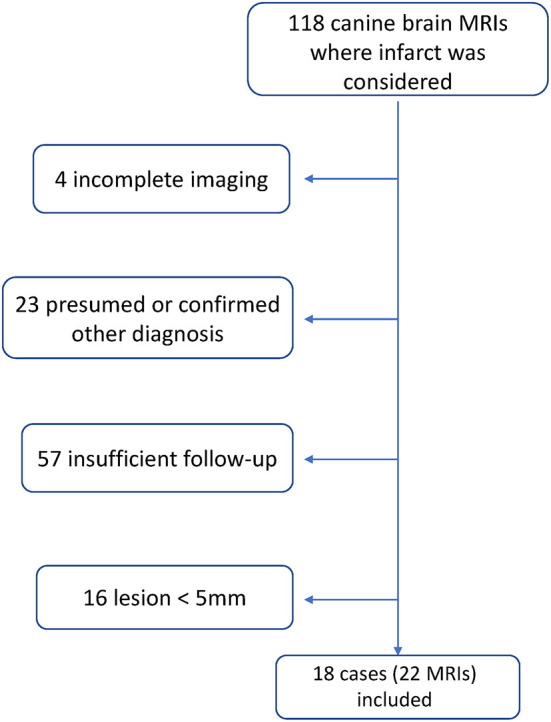
Schematic of record search and case selection process.

During the specified interval 1,546 canine brain MRIs were performed at the institution on 1,391 patients. Figure 1 shows the process of case identification. After sequential application of the parameters listed in the Methods, there remained 18 cases that met all inclusion criteria.

Of these, eight were presumed or confirmed ischemic CVA and ten were presumed or confirmed hemorrhagic CVA. Six of the ischemic CVA met the criterion of ≥ 5 months of clinical follow-up without progression of signs; two had histopathological confirmation. Five hemorrhagic CVA had ≥ 5 months of clinical follow-up without progression of signs; one had histopathological confirmation; and four had repeat MRI showing a static to resolving lesion, yielding a total of 22 image sets for analysis.

### Clinical data

No cases were excluded due to gradual onset of signs. Table 1 shows the signalment and time elapsed between onset of signs and MRI; with cases having repeat MRI represented twice. Ischemic lesion ages ranged from 0–5 days. Hemorrhagic lesion ages ranged from 0–132 days. Eight of the hemorrhagic CVA image sets had T2-FLAIR hyperintensity exclusive of the region of T2^*^ hypointensity. All of these had a lesion age of ≤ 14 days. Six hemorrhagic CVA image sets did not have T2-FLAIR hyperintensity beyond the bounds of the region of T2^*^ hypointensity; five of these had a lesion age of ≥21 days.

**Table 1:** Clinical data for 22 image sets included in this report.

**Age (yrs)**	**Breed**	**Sex/Neuter status**	**Duration of signs (days)**	T2*signal void	**EPI**	**Lesion location**	**Median ADC contralateral control (x10^−3^ mm^2^/s)**	**Median ADC T2-FLAIR ROI (x10^−3^ mm^2^/s)**	**Median relative ADC T2-FLAIR ROI**
12	Mixed breed	FI	0	N	N	medulla	0.631	0.300	0.475
13	Yorkshire terrier	MN	0	N	Y	cerebellum	0.765	0.399	0.522
12	Greyhound	FN	1	N	N	thalamus + midbrain	0.580	0.888	1.53
1	Labrador retriever	FI	2	N	N	internal capsule	0.757	0.757	1.00
12	Shih Tzu	FN	2	N	Y	midbrain	0.739	0.436	0.590
16	Bichon Frise	MN	3	N	Y	cerebellum	0.944	0.729	0.791
14	Chihuahua	MN	3	N	N	midbrain + cerebellum	0.633	0.582	0.919
5	Cavalier King Charles spaniel	FN	4	N	N	cerebellum	0.677	0.676	0.999
3	Pit bull terrier	M	0	Y	N	subcortical white matter	0.448	0.990	2.21
11	Mixed breed	MN	2	Y	N	subcortical white matter	0.672	1.27	1.88
6	Boston terrier	MN	2	Y	Y	subcortical white matter	0.485	1.31	2.69
4	Pit bull terrier	FN	3	Y	Y	subcortical white matter	0.706	0.933	1.32
12	Mixed breed	FN	3	Y	Y	subcortical white matter	0.699	1.27	1.81
15	Cardigan Welsh Corgi	FN	5	Y	N	frontal cortex	0.756	n/a	n/a
11	Yorkshire terrier	FN	5	Y	N	subcortical white matter	0.673	1.23	1.83
	*2nd MRI*		32	Y	N	subcortical white matter	0.641	n/a	n/a
9	Golden retriever	MN	11	Y	Y	subcortical white matter	0.721	0.857	1.19
	*2nd MRI*		65	Y	Y	subcortical white matter	0.754	n/a	n/a
11	Australian cattle dog	FN	14	Y	N	subcortical white matter	0.684	1.11	1.62
	*2nd MRI*		132	Y	N	subcortical white matter	0.692	n/a	n/a
13	Australian shepherd	FN	27	Y	N	occipital cortex	0.885	n/a	n/a
	*2nd MRI*		132	Y	N	occipital cortex	1.14	n/a	n/a

Column labeled T2^*^ signal indicates hemorrhagic (Y) or ischemic (N) presumed CVA. Column labeled EPI indicates if echo planar (Y) or non-echo planar (N) diffusion-weighted imaging was used. Duration of clinical signs is the number of days elapsed between the onset of neurological signs and MRI. Because ADC values were only analyzed for those image sets that had T2-FLAIR hyperintensity on MRI, hemorrhagic presumed CVAs with no T2-FLAIR hyperintensity do not have ADC values.

### Median ADC values of normal control regions

Table 1 shows the location of each lesion and median ADC value of the contralateral matched parenchyma. Ischemic CVAs occurred in the brainstem and cerebellar gray matter. Hemorrhagic CVAs occurred in the forebrain, and the associated T2-FLAIR hyperintensity was seen predominantly in subcortical white matter. Normal control median ADC values were in general < 1.0x10^−3^ mm^2^/s (see Discussion), with lower values for brainstem structures and subcortical white matter, and slightly higher values for cerebellar gray matter and cortical tissue.

### DWI of T2-flair hyperintense regions

**Figure 2:**
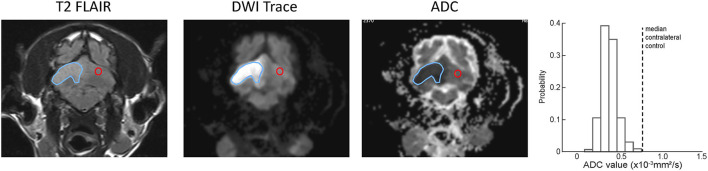
Examples of ROI (blue outline) and contralateral normal control (red outline) region selection for a single slice from representative example ischemic CVA. A region was selected for every slice on the T2-FLAIR transverse images and copied to the corresponding slice for the DWI and ADC series. The histogram at the right shows the ADC values within the ROI, as well as the median ADC value within the contralateral control region (dotted line).

Figure 2 shows selected transverse images demonstrating a ROI and the corresponding contralateral normal control region superimposed on the DWI and ADC series for a representative ischemic CVA. The probability histogram for pixel values within the ROI, with the median value of the contralateral normal control region also indicated, are shown for this example.

**Figure 3:**
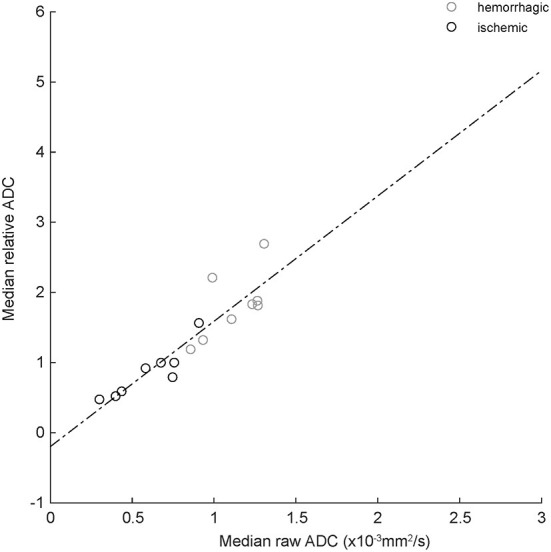
Correlation between absolute and relative median ADC values for ischemic (black circles) and hemorrhagic (gray circles) CVAs. The dashed line is best-fit linear regression (equation in text). R^2^ = 0.82, indicating good correlation.

Figure 3 shows the relationship between median absolute and relative (normalized to the median value of the contralateral normal control region) ADC values for the eight ischemic and eight hemorrhagic CVAs that exhibited T2-FLAIR hyperintensity outside of the region of T2^*^ hypointensity (see Materials and Methods). The best fit regression line is represented by *ADC*_*relative*_= *1.79* ^*^ *ADC*_*raw*_*-0.2*.

**Figure 4:**
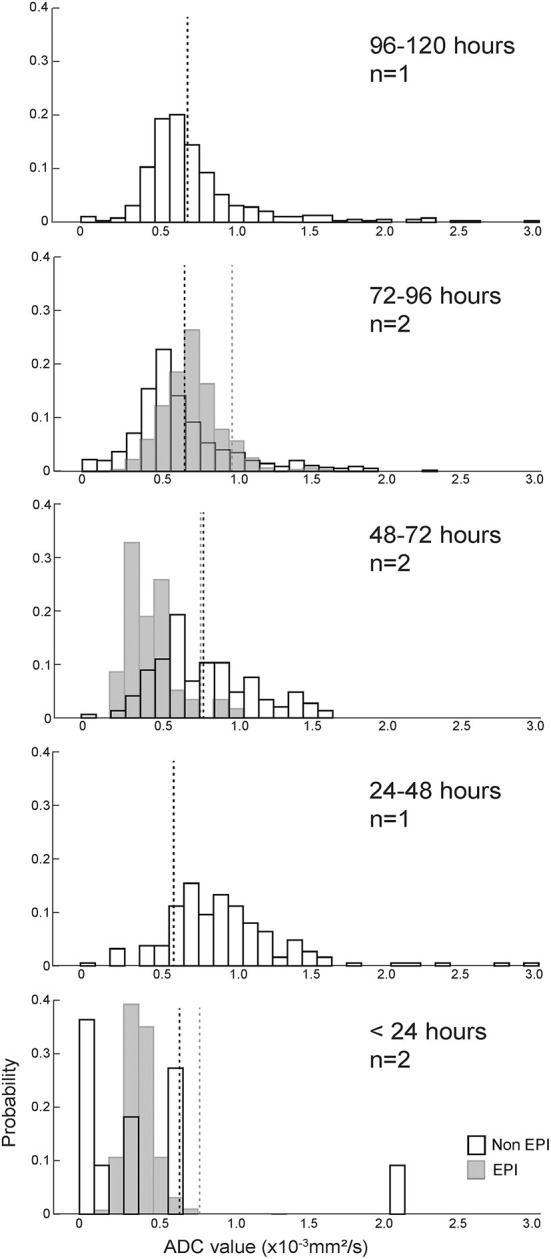
ADC value histograms for eight ischemic CVAs, arranged by duration of clinical signs (more recent events below older events). Black outline bars indicate non-EPI DWI, and light gray bars indicate EPI DWI. The dotted lines show median ADC values for the control regions for each histogram, with black dotted lines corresponding to non-EPI DWI, and gray dotted lines corresponding to EPI DWI. Median absolute and relative ADC values for the T2-FLAIR ROIs, median absolute ADC values for the contralateral control regions, and anatomical locations of the lesions/control selections are listed in Table 1. For most cases, the ROIs had median absolute ADC values < 1.0x10^−3^ mm^2^/s and median relative ADC values < 1. The CVAs with the shortest duration had the lowest ADC values.

Figure 4 shows the probability histograms for the absolute and relative pixel values on ADC series for the eight ischemic CVA in our data set, arranged by lesion age. The median absolute and relative ADC value for each scan is shown in Table 1. The median absolute ADC values were lowest for the two ischemic CVA that were imaged within 24 h of the onset of signs.

**Figure 5:**
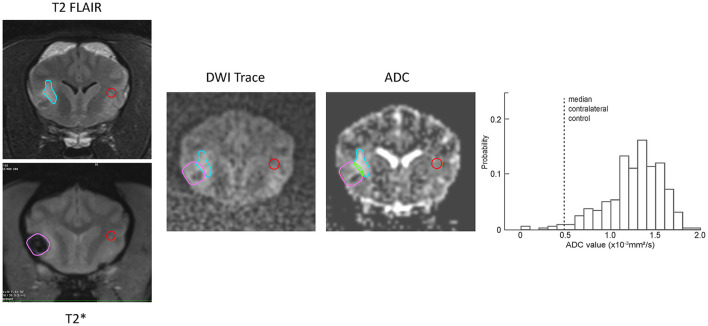
Examples of ROI (blue outline) and contralateral normal control (red outline) region selection for a representative example hemorrhagic CVA. The regions were selected on the T2-FLAIR transverse images and copied to the DWI and ADC series. The region of T2* hypointensity was defined on the corresponding T2* transverse image (lavender outline). Pixels that were within both the T2-FLAIR hyperintense region and the T2* hypointense region (green outline) were excluded from the resulting probability histogram (right). The histogram shows the ADC values of non-excluded pixels within the ROI, as well as the median ADC value within the contralateral control region (dotted line).

**Figure 6:**
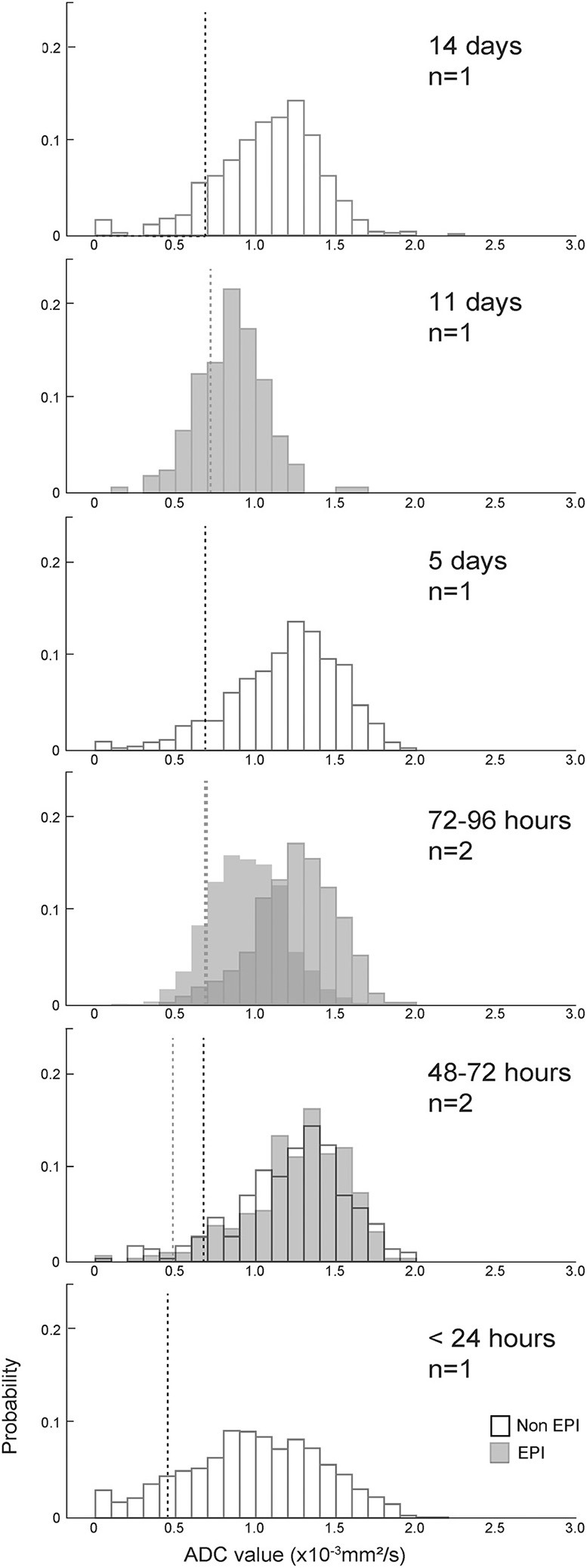
ADC value histograms for the T2-FLAIR hyperintense region adjacent to eight hemorrhagic CVAs, arranged by duration of clinical signs (more recent events below older events). Black outline bars indicate non-EPI DWI, and gray bars indicate EPI DWI. The dotted lines show median ADC values for the control regions for each histogram, with black dotted lines corresponding to non-EPI DWI, and gray dotted lines corresponding to EPI DWI. Median absolute and relative ADC values for the T2-FLAIR ROIs, median absolute ADC values for the contralateral control regions, and anatomical locations of the lesions/control selections are listed in Table 1. There was no evidence of restricted diffusion adjacent to the T2* hypointense region in any dog.

For the corresponding analysis of hemorrhagic CVA, ADC values associated with regions of T2-FLAIR hyperintensity were defined to exclude pixels that were also within the region of T2^*^ hypointensity. Figure 5 shows an example of the process for generating the probability histogram for an example hemorrhagic CVA. Figure 6 shows the probability histograms for the T2-FLAIR hyperintense regions of the eight hemorrhagic CVA that had such regions, arranged by lesion age, analogous to Figure 4. The median absolute and relative ADC value, and corresponding median control ADC value, for each scan is shown in Table 1. The median absolute ADC values for these lesions were generally greater than the median ADC value of the contralateral control region, and >1.0x10^−3^ mm^2^/s, for all lesion ages.

### DWI of T2^*^ hypointense regions

Five of the hemorrhagic CVA image sets were generated using proprietary EPI DWI (RESOLVE), with b = 1000s/mm^2^. Nine of the hemorrhagic CVA image sets were generated using proprietary non-EPI DWI (BLADE), with b = 800s/mm^2^. See Figure 5 for an example of selected transverse images demonstrating a ROI, identified on T2^*^ transverse images, superimposed on the (EPI) DWI and ADC series for a representative hemorrhagic CVA.

It has been suggested that the greater susceptibility artifact in EPI DWI may artificially lower ADC values near paramagnetic regions (such as areas of hemorrhage). To determine if EPI vs. non-EPI DWI had a systematic impact on ADC values calculated within a hemorrhagic region, we evaluated the distribution of pixel values that lay within the bounds of the hypointense region defined on corresponding T2^*^ series for the 14 hemorrhagic CVA image sets, segregated by method of image generation. Pixels within the T2^*^ hypointense region were more likely to have a value of ADC=0 on non-EPI DWI sets (25.8% vs. 13.6%). ADC value is calculated as


(1) $$\begin{array}{l} \text{ADC}=-ln\left(\text{SI}{}_{b0}/\text{SI}{}_{b1}\right)/\left(b0-b1\right) \end{array}$$

**Figure 7:**
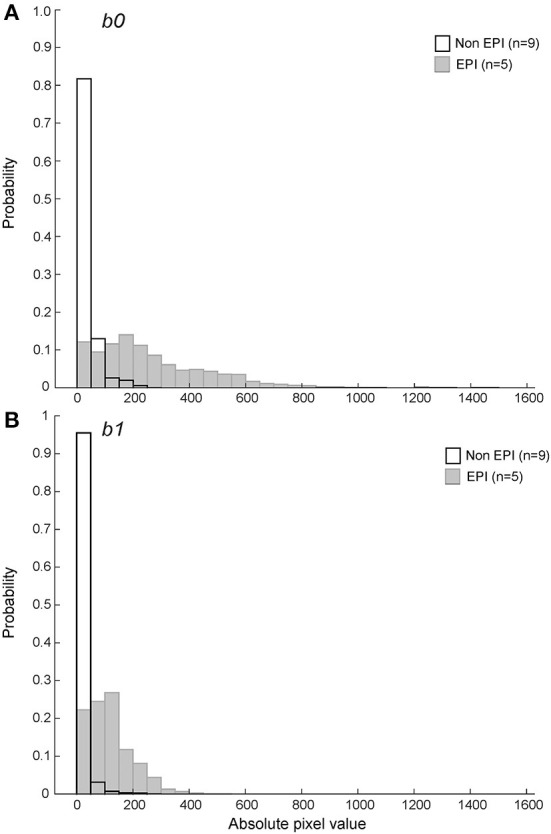
Probability histograms of pixel values within the T2* hypointense ROIs for *b0* **(A)** and *b1* **(B)** DWI series (see text for variable definitions). Non-EPI series used *b1* = 800s/mm^2^ and are shown in black outline. The pixel values within ROIs defined by the T2* hypointense region were more likely to be zero or close to zero on non-EPI ADC series for both *b0* and *b1*.

Where, *SI*_*b*0_ is the pixel intensity value at *b0, SI*_*b*1_ is the pixel intensity at *b1, b0* = 0s/ mm^2^, and *b1* = 800s/mm^2^ for non-EPI and *b1* = 1000s/mm^2^ for EPI (see Materials and Methods for additional sequence details). The proportion of very low pixel values was greater for non-EPI DWI at both *b0* and *b1* (Figure 7).

## Discussion

DWI MRI characteristics are known to vary with hardware (36, 37) and software (37–39) properties and are subject to interobserver variability in measurement (40, 41). Nonetheless, they are included in many standard brain MRI series and are regularly interpreted by neuroradiologists and neurologists. In human neuroimaging, DWI is used to detect and characterize alterations in water diffusion as distinct from pathological processes that increase water content, and therefore T2-weighted signal intensity, without restricting diffusion. Major applications of this technique include improving sensitivity for detection, determination of onset, and estimation of lesion extent in CVAs (42–45). There are little existing published data on the DWI characteristics of spontaneous CVAs in dogs to inform appropriate clinical interpretation of these sequences.

### Absolute vs. relative ADC values

Consistent with previous reports (46, 47), we found that median ADC values for normal tissues in our sample population were generally < 1.0x10^−3^ mm^2^/s, indicating that identification of values below that cutoff within an ROI cannot be used in isolation to confirm pathologically restricted diffusion. A previous publication has demonstrated that absolute ADC values vary with anatomical location in normal dog brain (46), suggesting that relative ADC values may be a better tool for identification of abnormal parenchyma.

### Ischemic CVAs

Previous investigations of an experimental ischemic stroke model using induced middle cerebral artery occlusion in healthy dogs showed relatively low ADC within the injured area at 3 days compared to 10 days (25) and high ADC values at 8 and 35 days (48). Because ADC for a given pixel is calculated based on the relationship between the *SI*_*b*0_ and *SI*_*b*1_ value (see Equation 1 in *Results*), an increase in ADC may occur due to a decrease in *SI*_*b*1_, and increase in *SI*_*b*0_, or both. In both referenced studies, the increase in ADC over time was shown to correlate with a decline in *SI*_*b*1_ pixel values within the injured region, with little change in T2-weighted *SI*_*b*0_ or T2-FLAIR signal within the region over the same timepoints.

In humans, ADC values have been reported to remain reduced relative to normal values in ischemic stroke for ≥ 1 week (10, 12), with a transition period between 8 and 14 days (10), and increased ADC values after 14 days (10) and at 30 days (12). Others have demonstrated that the time course of evolution of ADC values after ischemic stroke in humans can be affected by recovery of perfusion (49), further complicating interpretation in lesions for which the exact time of onset is unknown.

Reports of ADC values in spontaneous canine CVAs are sparse, especially those using within-patient control values. One case series identified two ischemic CVAs with estimated age of 24–48 h and absolute median ADC values of 0.67 and 0.68 x 10^−3^ mm^2^/s, and one ischemic CVA with estimated age of 10–14 days with absolute median ADC value of 1.10x10^−3^ mm^2^/s (14). Others indicate that the majority of presumed ischemic CVAs imaged 1–5 days after onset of signs had low ADC values (15, 16), though methods of calculation, values, and specific lesion ages were not reported.

Rodent ischemic CVA DWI abnormalities resolve faster than those observed in humans (4, 9, 31, 50). Dogs and primates are more gyrencephalic, have more gray matter volume, and have a larger proportion of subcortical white matter affected by induced stroke, as compared to rodents (50). Consistent with previous reports, we show examples of presumed canine CVAs with reduced ADC values up to 5 days after the onset of signs. The most profound reduction in ADC values was observed in the cases with the shortest duration of clinical signs, as expected by analogy to the evolution of ADC map changes in human CVAs.

### Hemorrhagic CVAs

The MRI appearances of presumed or confirmed naturally occurring intraparenchymal hemorrhage in dogs (51, 52) have been described in case series, but these do not include discussion of DWI characteristics. A single case series reports the absolute median ADC values for two hemorrhagic CVAs of 2–5 days as 2.19 and 1.10x10^−3^ mm^2^/s (14). It is not clear if the hemorrhagic region was excluded from the ROI.

As in a recent case series (51), many hemorrhagic CVAs in our case series had T2-FLAIR hyperintensity surrounding the hemorrhagic region. All lesions with such hyperintensity were imaged within 2 weeks of the onset of signs, whereas no lesions older than 3 weeks had surrounding T2-FLAIR hyperintensity; in presumed hemorrhagic CVAs that were re-imaged, previously noted T2-FLAIR hyperintensity had resolved. This, as well as subjective evaluation of the pattern of the hyperintensity as following subcortical white matter, is consistent with the interpretation of the T2-FLAIR hyperintensity surrounding the hemorrhagic region as transient vasogenic edema (51, 53). We did not find evidence of decreased relative ADC values within the T2-FLAIR hyperintense regions surrounding presumed hemorrhagic CVA, which may suggest mechanisms other than hemorrhagic transformation of ischemic infarct in these cases (54).

A previous experimental study of induced intracerebral hemorrhage in dogs (26) examined T2-FLAIR, DWI, and ADC imaging characteristics up to 24 days after lesion creation. The distribution and time course for T2-FLAIR hyperintensity in the model resembles that observed in our sample population. They likewise did not identify regions of reduced ADC values consistent with restricted diffusion beyond the margins of the hemorrhagic lesion.

### EPI vs. non-EPI DWI

EPI DWI uses a single excitation and multiple echoes in a train to fill all of k-space rapidly. Single-shot images are marred by marked susceptibility artifact, due to off-resonance shifts of precession during the echo train that cannot be compensated by shimming. Additionally, image spatial resolution is limited, and images are prone to geometric distortion (shrinking, scaling, shear) from uncompensated in-plane background gradients. Readout-segmented EPI (RESOLVE) is a Siemens proprietary multishot EPI technique that relies on parallel imaging. The multiple shots allow a single image to be formed in k-space from data acquired in two or more RF excitations, shortening the readout interval; however, the number of excitations is still lower than that in non-EPI DWI. The parallel imaging technique also infers lines of k-space during initial filling, shortening total readout duration further, which reduces the accumulation of off-resonance effects during the echo train.

Non-EPI DWI fills only a portion of k-space with data obtained from a single excitation. This allows for higher spatial resolution and less susceptibility artifact than in EPI DWI. Originally conceived as a single line of k-space per excitation, other non-EPI k-space filling schemes (e.g., radial filling as used by the Siemens proprietary non-EPI DWI sequence BLADE) have been developed to oversample the central region of k-space (representing the highest-amplitude signals), which improves signal-to-noise ratio further and allows for motion correction between excitations. Image averaging after motion correction is also employed to improve signal-to-noise ratio. However, the large number of excitations and the repeated sampling required for image averaging both increase scan time for these sequences. Additionally, the motion correction algorithms can introduce new artifacts (55) in some circumstances.

Although several publications have reported DWI characteristics of spontaneous hemorrhagic lesions in humans (11, 13, 56, 57), the clinical interpretation of these findings is complicated by susceptibility effects of paramagnetic blood products on T2-weighted image sequences, including those used in DWI. Specifically, the problem of “T2 blackout,” where susceptibility artifact causes very low pixel values in the vicinity of the paramagnetic substance, may artificially lower the pixel values on DWI within hematomas. Because EPI is considered more susceptible to paramagnetic distortion of the local magnetic field, we anticipated more evidence of T2 blackout around hemorrhagic CVAs imaged with EPI techniques than non-EPI techniques. However, we found that pixel values near zero within the T2^*^ hypointense region were more commonly seen with non-EPI DWI, possibly as a manifestation of the lower signal-to-noise ratio in these series.

As with most retrospective clinical case series, our report is limited by the small number of cases suitable for inclusion, additional noise introduced by variability in imaging parameters used, and the presumption of diagnosis in patients with good outcomes. For hemorrhagic CVAs, there is the additional challenge of defining the hemorrhagic area, given that susceptibility artifact “blooms” from the source of the magnetic field inhomogeneity and is larger than the actual region of hemorrhage. These challenges are likely contributing factors to the overall lack of robust publications on DWI features of spontaneous canine CVAs. This report demonstrates the variability in ADC values for lesions that have the clinical behavior of CVAs, suggesting that CVA cannot necessarily be excluded as a differential solely on the absence of evidence of restricted diffusion, especially for lesions imaged beyond the acute timeframe. As with human CVAs, temporal evolution of perfusion after initial injury may influence the DWI appearance of canine CVAs, though these data are too sparse to serve as the basis for a temporally-specific reference range. Regarding canine hemorrhagic CVAs, the absence of evidence of restricted diffusion in the perilesional T2-FLAIR hyperintense regions suggests that hemorrhagic transformation of ischemic infarction is not a common etiology of solitary spontaneous intracranial hemorrhage in dogs in our sample population. Further, it may indicate that canine models of intracerebral hemorrhage (26) reflect imaging characteristics of the spontaneous clinical disease. Finally, we did not identify a clear advantage of non-EPI over EPI DWI for the characterization of presumed CVAs, with or without hemorrhage, on our 3T magnet. Specifically, EPI was not more likely to exhibit signal dropout within or immediately surrounding a region of hemorrhage (defined by the area of T2^*^ hypointensity) than non-EPI in our case set.

To better characterize the temporal evolution of presumed spontaneous CVAs in dogs, prospectively collected serial MRI at predefined timepoints after the onset of neurological signs would be needed. To determine optimal parameters for detection of restricted diffusion in canine CVAs, a systematic exploration of the space defined by echo generation method, b-value, magnet strength, echo and repetition timing, and coil selection would be necessary. To determine the reliability of ADC calculations in this population of dogs, evaluation of agreement across and within observers would be necessary. It is likely that a combination of diffusion and perfusion-weighted imaging techniques, as is currently standard-of-care in human cerebrovascular imaging, would further improve our understanding of the evolution of parenchymal damage and recovery in canine CVA.

## Data availability statement

The original contributions presented in the study are included in the article/Supplementary material, further inquiries can be directed to the corresponding author.

## Author contributions

JL and JG contributed to concept and study design. SK and ED contributed to data collection. EB contributed to data analysis and initial manuscript preparation. All authors contributed to manuscript editing and refinement.

## Conflict of interest

The authors declare that the research was conducted in the absence of any commercial or financial relationships that could be construed as a potential conflict of interest.

## Publisher's note

All claims expressed in this article are solely those of the authors and do not necessarily represent those of their affiliated organizations, or those of the publisher, the editors and the reviewers. Any product that may be evaluated in this article, or claim that may be made by its manufacturer, is not guaranteed or endorsed by the publisher.
