# Supplementary material for: Temporal and sequence-related variability in diffusion-weighted imaging of presumed cerebrovascular accidents in the dog brain
Source: Front Vet Sci. 2022 Nov 7;9:1008447. doi: 10.3389/fvets.2022.1008447 (PMC9676236; doi:10.3389/fvets.2022.1008447)
Supplement: Supplementary file 1 [file Table_1.docx]

|  | T2-FLAIR | T2* | BLADE (non-EPI) | RESOLVE (EPI) |
| --- | --- | --- | --- | --- |
| TE (ms) | 79 - 95 | 19.9 | 130 | 62 - 130 |
| TR (ms) | 5800 - 9000 | 660 - 954 | 8840 - 10040 | 6120 - 8840 |
| Slice thickness (mm) | 2 - 2.5 | 2 - 2.5 | 2.5 | 2.2 - 3 |
| b-value (s/mm^2^) | - | - | 800 | 1000 |
| FOV (mm) | 113 x 125 | 113 x 125 | 125 x 125 | 125 x 125 |
| Acquisition matrix | 464 x 512 | 174 x 192 | 128 x 128 | 128 x 128 |
| Scan time (min:sec) | 4:30 | 3:41 | 6:38 | 5:29 |

**Supplemental Table 1.** Minimum and maximum parameter settings for the analyzed MRI sequences. A Siemens 15-channel transmit/receive knee coil was used for all acquisitions.
